# Medical Evidence of Human Rights Violations against Non-Arabic-Speaking Civilians in Darfur: A Cross-Sectional Study

**DOI:** 10.1371/journal.pmed.1001198

**Published:** 2012-04-03

**Authors:** Alexander C. Tsai, Mohammed A. Eisa, Sondra S. Crosby, Susannah Sirkin, Michele Heisler, Jennifer Leaning, Vincent Iacopino

**Affiliations:** 1 Robert Wood Johnson Health and Society Scholars Program, Harvard University, Cambridge, Massachusetts, United States of America; 2 Center for Global Health, Massachusetts General Hospital, Boston, Massachusetts, United States of America; 3 Physicians for Human Rights, Cambridge, Massachusetts, United States of America; 4 Harvard Humanitarian Initiative, Harvard University, Cambridge, Massachusetts, United States of America; 5 Department of Medicine, Boston University School of Medicine, Boston, Massachusetts, United States of America; 6 Department of Health Law, Bioethics, and Human Rights, Boston University School of Public Health, Boston, Massachusetts, United States of America; 7 Veterans Affairs Center for Clinical Management Research, Veterans Affairs Ann Arbor Healthcare System, Ann Arbor, Michigan, United States of America; 8 Department of Internal Medicine, University of Michigan Medical School, Ann Arbor, Michigan, United States of America; 9 Department of Health Behavior and Health Education, School of Public Health, University of Michigan, Ann Arbor, Michigan, United States of America; 10 Francois-Xavier Bagnoud Center for Health and Human Rights, Harvard School of Public Health, Boston, Massachusetts, United States of America; 11 Department of Medicine, University of Minnesota Medical School, Minneapolis, Minnesota, United States of America; 12 Human Rights Center, University of California at Berkeley, Berkeley, California, United States of America; London School of Hygiene and Tropical Medicine, United Kingdom

## Abstract

**Background:**

Ongoing conflict in the Darfur region of Sudan has resulted in a severe humanitarian crisis. We sought to characterize the nature and geographic scope of allegations of human rights violations perpetrated against civilians in Darfur and to evaluate their consistency with medical examinations documented in patients' medical records.

**Methods and Findings:**

This was a retrospective review and analysis of medical records from all 325 patients seen for treatment from September 28, 2004, through December 31, 2006, at the Nyala-based Amel Centre for Treatment and Rehabilitation of Victims of Torture, the only dedicated local provider of free clinical and legal services to civilian victims of torture and other human rights violations in Darfur during this time period. Among 325 medical records identified and examined, 292 (89.8%) patients from 12 different non-Arabic-speaking tribes disclosed in the medical notes that they had been attacked by Government of Sudan (GoS) and/or Janjaweed forces. Attacks were reported in 23 different rural council areas throughout Darfur. Nearly all attacks (321 [98.8%]) were described as having occurred in the absence of active armed conflict between Janjaweed/GoS forces and rebel groups. The most common alleged abuses were beatings (161 [49.5%]), gunshot wounds (140 [43.1%]), destruction or theft of property (121 [37.2%]), involuntary detainment (97 [29.9%]), and being bound (64 [19.7%]). Approximately one-half (36 [49.3%]) of all women disclosed that they had been sexually assaulted, and one-half of sexual assaults were described as having occurred in close proximity to a camp for internally displaced persons. Among the 198 (60.9%) medical records that contained sufficient detail to enable the forensic medical reviewers to render an informed judgment, the signs and symptoms in all of the medical records were assessed to be consistent with, highly consistent with, or virtually diagnostic of the alleged abuses.

**Conclusions:**

Allegations of widespread and sustained torture and other human rights violations by GoS and/or Janjaweed forces against non-Arabic-speaking civilians were corroborated by medical forensic review of medical records of patients seen at a local non-governmental provider of free clinical and legal services in Darfur. Limitations of this study were that patients seen in this clinic may not have been a representative sample of persons alleging abuse by Janjaweed/GoS forces, and that most delayed presenting for care. The quality of documentation was similar to that available in other conflict/post-conflict, resource-limited settings.

*Please see later in the article for the Editors' Summary*

## Abstract 2

Alexander Tsai and colleagues review medical records from the Amel Centre, Sudan, to assess consistency between recorded medical evidence and patient reports of human rights violations by the Government of Sudan and Janjaweed forces.

## Editors' Summary

**Background:**

Conflict in the Darfur region of Sudan between Arabic- and non-Arabic-speaking tribes over the past decade has resulted in a severe humanitarian crisis. According to the United Nations, more than 2.7 million people have fled from their homes to camps for internally displaced persons (IDPs) or to refugee camps in neighboring Chad, and up to 300,000 people have died from war, hunger, and disease since the conflict started. The origins of this conflict go back many years, but in 2003, organized rebel forces began attacking government targets, accusing the Government of Sudan (GoS) of oppressing black Africans in favor of Arabs. In response, the GoS attacked the rebel forces, but some observers allege it also targeted non-Arabic-speaking civilians, in contravention of international laws of war. Observers have also accused the GoS of having links with the Janjaweed militias, nomadic Arabs who attack settled black farmers, although the GoS denies any such links. Indeed, reports of systematic, targeted assaults on non-Arabic-speaking civilians, of large-scale disruption of rural livelihoods, and of deliberate consignment to living conditions likely to cause death have prompted some observers to accuse the GoS of genocide (violent crimes committed against a national, ethnical, racial, or religious group with the intention of destroying that group) and the International Criminal Court to issue arrest warrants for the allegedly responsible authorities.

**Why Was This Study Done?:**

Most investigations of claims of violence against civilians in Darfur have relied on self-reported data gathered from people living in refugee camps outside Sudan. Because these data could be biased, in this cross-sectional study (a study that characterizes a population at a single point in time), the researchers investigate the nature and geographic scope of alleged abuses against civilians in Darfur and endeavor to substantiate these allegations by analyzing the medical records of patients attending the Amel Centre for Treatment and Rehabilitation of Victims of Torture in Nyala, South Darfur. Opened in 2004, this center provided free clinical and legal services to civilians affected by human rights violations. Its staff fled in 2009 because of increasingly dangerous working conditions; the medical records used in this study were sent out of Sudan before the staff fled.

**What Did the Researchers Do and Find?:**

Between September 28, 2004, and December 31, 2006, 325 patients were seen at the Amel Centre. According to their medical records, 292 patients from 12 different non-Arabic-speaking tribes alleged that they had been attacked by GoS or Janjaweed forces in rural areas across Darfur. Nearly all the patients reported that they had been attacked in the absence of active armed conflict between GoS/Janjaweed forces and rebel groups. Half of them claimed that they had been beaten, two-fifths reported gunshot wounds, a third reported destruction or theft of property, and nearly a third reported involuntary detainment. Half of the 73 women seen at the center disclosed that they had been sexually assaulted, often near IDP camps. Only 198 medical records contained sufficient detail to enable the researchers to determine whether the documented medical evidence was consistent with the alleged abuses. However, in all these cases, the researchers judged that the medical evidence was consistent with, highly consistent with, or virtually diagnostic of the alleged abuses.

**What Do These Findings Mean?:**

These findings provide credible medical evidence that is consistent with torture and other human rights violations being inflicted on non-Arabic-speaking civilians in Darfur from 2004 to 2006. These findings cannot be used to estimate the population incidence of attacks on civilians or to corroborate claims of assailants' identities or of genocidal intent. Moreover, their accuracy may be affected by several limitations of this study. For example, during the study period, only patients who obtained a medical evidence form from the police were permitted to receive treatment from an authorized medical officer; obtaining such a form likely represented a considerable hurdle to accessing health care services. Nevertheless, the widespread, organized, and sustained pattern of attacks documented in this study is consistent with the possibility that the actions of Janjaweed and GoS forces during the conflict in Darfur may constitute war crimes, crimes against humanity, and/or acts of genocide. Importantly, these findings also highlight the need to provide adequate protection for health professionals working in countries affected by internal conflicts.

**Additional Information:**

Please access these web sites via the online version of this summary at http://dx.doi.org/10.1371/journal.pmed.1001198.

- The African UnionUnited Nations Mission in Darfur (UNAMID) provides background information and up-to-date news about the ongoing conflict in Darfur Amnesty International, which campaigns for human rights, provides background information and news about the current situation in Darfur
- The Save Dafur Coalition also provides detailed information about the situation in Darfur Physicians for Human Rights, a non-profit organization that mobilizes health professionals to advance health, dignity, and justice, is calling for security in Darfur and compensation and restitution for survivors of the conflict
- Wikipedia has pages on Darfur and on genocide (note that Wikipedia is a free online encyclopedia that anyone can edit; available in several languages)
- Details on warrants of arrest issued by the International Criminal Court in response to the situation in Dafur are available

## Introduction

In the Darfur region of western Sudan, ongoing conflict between Arabic-speaking and non-Arabic-speaking tribes [1],[2] has reached crisis proportions since the Government of Sudan (GoS) first initiated its military response to organized armed groups opposing the GoS [2]. In addition to targeting armed rebel forces in its response, however, the GoS has also been accused of targeting non-Arabic-speaking civilians, namely members of the Fur, Masalit, and Zaghawa tribes [3],[4]. By the end of 2007, more than 2.4 million refugees from the violence, or nearly one-third of the population [5], had fled to camps for internally displaced persons (IDPs) within Darfur or to similar refugee camps in neighboring Chad [6], thus creating a severe humanitarian disaster.

Prior research has focused on generating accurate mortality estimates to inform policy and programming [7]–[13], with recent studies estimating 200,000–300,000 deaths directly and indirectly attributable to the conflict in 2003–2005 alone [14],[15]. The reported systematic, repeated, targeted assaults on non-Arabic-speaking civilians, large-scale disruption of rural livelihoods, and deliberate consignment to conditions conducive to death have prompted observations that these could constitute acts of genocide [4],[5],[16]–[19]. Following a United Nations–appointed Commission of Inquiry and an International Criminal Court (ICC) investigation, the Pre-Trial Chamber I of the ICC issued arrest warrants for allegedly responsible authorities, including two arrest warrants for Sudanese President Omar Hassan Ahmad Al Bashir (“Al Bashir”) on the grounds of crimes against humanity (March 4, 2009) [20],[21] and genocide (July 12, 2010) [22].

Despite investigations into the violence in Darfur, little research to date has been able to make use of Sudanese documents to substantiate victims' or observers' claims of violence amounting to war crimes, crimes against humanity, or genocide. GoS forces were implicated in the Atrocities Documentation Survey [5],[23]–[25]. Arab Janjaweed (“men with guns on horses or camels”) militias, which originated as Libyan proxy militias in the Chadian civil war and have been suspected of collaborating with GoS forces [2], have been implicated in reports of sexual violence described by Darfuri women now living in IDP camps [26]. The systematic destruction of livelihoods, which under certain circumstances can be considered an act of genocide [17],[27],[28], has also been described. However, a critical limitation of prior studies is their reliance on self-report data gathered from victims living in refugee camps outside of Sudan. One team of investigators attempted to conduct interviews at IDP camps within Darfur but was refused access by the GoS [17],[27]. With unique access to medical records of clinical encounters in Darfur, we undertook this study to characterize the nature and geographic scope of alleged abuses against civilians in Darfur and to substantiate the allegations with forensic review and analysis of the medical evidence.

## Methods

### Ethics Statement

As this was a retrospective analysis of de-identified medical records, informed consent was not obtained. All study procedures were approved by the Harvard School of Public Health Office of Human Research Administration as well as an independent ethics review board convened for this research project by Physicians for Human Rights. Given that the medical records used in the analysis were de-identified, this research project was assessed to represent minimal risk to Amel Centre patients. The ethics review board was guided by the relevant process provisions of Title 45 of the US Code of Federal Regulation and the Declaration of Helsinki as revised in 2000 [29] and was composed of individuals with expertise in forensic medicine, public health, bioethics, and international health and human rights research.

### Study Population and Setting

Data for this study consisted of 325 de-identified medical records of all initial visits (i.e., excluding follow-up visits) of patients seen for treatment at the Amel Centre for Treatment and Rehabilitation of Victims of Torture, in Nyala, South Darfur, from its opening on September 28, 2004, through December 31, 2006. Records for 2007–2009 could not be retrieved because of ongoing security concerns (as described in more detail below). With funding from the European Commission, the United Nations High Commissioner for Refugees, the US Agency for International Development, and the United Nations Development Programme, the Amel Centre was the only dedicated local non-governmental provider of free clinical and legal services to any civilian victim of torture or other human rights violations. The Amel Centre received referrals from volunteers placed in three large IDP camps near Nyala (Dreig, Otash, and Kalma) but accepted civilian clients from all over Darfur. Aside from the free services, and transportation to and from the IDP camps, patients were not given additional incentives or benefits.

The Amel Centre's initial staff in the Nyala office consisted of one general medicine doctor (M. A. E.), one junior doctor, one psychosocial worker, and two lawyers. Their training on the treatment of victims of torture and sexual violence was facilitated by the Sudan Organisation Against Torture and was consistent with the *Manual on Effective Investigation and Documentation of Torture and Other Cruel, Inhuman or Degrading Treatment or Punishment* (“Istanbul Protocol”) [30]–[32]. The paper-based record-keeping system was similar to other prototypical clinics operating in conflict zones. Although examining clinicians typically conversed with patients in the patients' language of choice (typically Fur, Zaghawa, or Dago), notes documenting the encounters were written in English. A standardized medical record form was used, but few fields specified closed-ended responses (e.g., “name,” “date of birth,” “date of detention”). The content of the clinical encounter, and therefore the bulk of the medical record documentation, was driven by patients' concerns. A network of volunteer physicians and social workers provided specialty care, and all women who disclosed that they had been sexually assaulted were referred to a gynecologist for evaluation. The laboratories associated with the network were able to implement all necessary blood, urine, stool, serological, and pregnancy tests but did not have the capacity for deoxyribonucleic acid analysis. After the initial visit, follow-up visits were provided to assess symptomatic improvement and provide longer term physiotherapy where indicated. The care provided and coordinated by the Amel Centre was delivered under difficult and often dangerous field conditions. After the ICC issued the first arrest warrant for Al Bashir [21], the Sudanese Ministry of Humanitarian Affairs ordered the Amel Centre, along with two other local and 13 foreign aid groups, to cease operations [33]. The three clinicians formerly on staff are now living abroad. Prior to fleeing the country, they preserved hard copies of the medical records for 2004–2006 and sent them out of the country.

### Data Collection

Amel Centre clinicians generated the medical records for the purposes of clinical care and internal record-keeping. We sought to abstract the data both accurately and in such a way as to capture the main themes identified in the records. Guided by prior research [34]–[41], we created a list of different types of abuses that may be considered evidence of torture and/or other human rights violations, as well as symptoms potentially reported by patients and signs potentially documented by examining clinicians. Then we developed a medical record abstraction tool that included lists of standardized names (e.g., tribes and rural council areas) and response options to guide efficient abstraction of data (Text S1).

Data were abstracted by one of the authors (A. C. T.) from the demographic, incident, and clinical care components of the Amel Centre's general medical records. Although the general medical records may have noted the presence of diagnostic or laboratory testing, or specialty medical records, access to these raw data elements unfiltered by the examining clinicians (e.g., diagnostic imaging or laboratory reports) were not available for our analysis. We collected data on patient socio-demographic characteristics, alleged abuses, and the harms reportedly resulting from the abuses. To assess the reliability of the data abstraction tool for coding the key variables on alleged abuses, we randomly selected 20 medical records for independent coding by two other study authors (M. A. E. and S. S. C.) as well as for wider discussion by the research team. Inter-rater agreement exceeded 0.70 on the coding of most of these variables. The abstraction tool was further refined through an iterative process to ensure that the variables were clearly defined and could be applied consistently to the data. With regards to content validity, the 20 records were carefully reviewed to ensure that all potential categories were represented. Data from the remaining 305 medical records were then abstracted by a single author (A. C. T.).

### Medical Opinions on Alleged Abuses

Two study authors with extensive medical experience in the evaluation and treatment of survivors of torture and other forms of physical and psychological abuse (S. S. C. and V. I.) independently reviewed each medical record, blinded to each other's assessments. First, they determined whether the medical record contained sufficient detail to enable an informed opinion about the consistency of the documented signs and symptoms with the record of alleged abuses in the medical notes. Second, among the cases that did contain sufficient detail, they assessed the extent to which the recorded signs and symptoms were consistent with the alleged abuses described in the medical record. Consistency was scored using a five-point Likert-type scale: “not related to alleged abuse,” “not consistent with,” “consistent with,” “highly consistent with,” and “virtually diagnostic of.” These evaluations were based on the Istanbul Protocol [30]–[32] and other conventions for the evaluation of survivors of torture and other human rights abuses [42]–[45].

### Statistical Analysis

Data were entered into Excel (version 12.0, Microsoft) and then exported to Stata (version 11.0, StataCorp) for analysis. We characterized socio-demographic, incident, and clinical variables with medians and inter-quartile ranges. Inter-rater agreement was assessed using the kappa statistic [46]. The locations of alleged attacks were mapped to the longitude and latitude [47]–[49] of the administrative center, principal town, or largest secondary town of the rural council area where they were reported to have taken place. ArcGIS (version 9.2, Esri) was used to generate a continuously variable proportional circle map, with circle sizes classified into seven categories using the Fisher-Jenks algorithm [50].

## Results

### Characteristics of Amel Centre Patients

We obtained medical records for all 325 patients presenting for care at the Amel Centre from September 28, 2004, to December 31, 2006. Summary statistics are presented in Table 1. Most patients were brought in by friends or relatives (54.2%) or by staff or volunteers (28.0%). Median age was 35 y, with a range of 4–82 y. Thirty patients (9.2%) were under the age of 18 y. Men comprised the majority of patients (252 [77.5%]). Approximately one-half (49.5%) of the men and boys were younger than 36 y of age. Most patients were married (76.3%). All self-identified as Muslim. The sample included patients from 14 different non-Arabic-speaking tribes, and members of the Fur, Zaghawa, and Dago tribes accounted for nearly 90% of patients. Only two (0.6%) patients were from Arab tribes. Most (84.9%) lived in South Darfur, where the Amel Centre was located.

**Table 1:** Characteristics of patients presenting for care at the Amel Centre for Treatment and Rehabilitation of Victims of Torture in Nyala, South Darfur.

Variable Name	Number (Percent)
**Referral source**	
Brought to center by friend/relative	176 (54.2%)
Brought to center by staff/volunteer	91 (28.0%)
Self-referral	44 (13.5%)
Referred by friend/relative	13 (4.0%)
**Visit year**	
2004	47 (14.5%)
2005	233 (71.7%)
2006	45 (13.9%)
**Sex**	
Male	252 (77.5%)
Female	73 (22.5%)
**Age**	
<26 y	96 (29.5%)
26–35 y	85 (26.2%)
36–45 y	68 (20.9%)
46–55 y	43 (13.2%)
>55 y	32 (9.9%)
**Marital status**	
Single	77 (23.7%)
Married	248 (76.3%)
**Has children**	
Yes	173 (53.2%)
No	3 (0.9%)
Unknown/unspecified	149 (45.9%)
**Religion**	
Muslim	325 (100%)
Other	0
**Tribe**	
Fur	173 (53.2%)
Zaghawa	76 (23.4%)
Dago	38 (11.7%)
Bargo	7 (2.2%)
Other	31 (9.5%)
**Occupation/profession**	
Farmer	199 (61.2%)
Student	42 (12.9%)
Merchant	26 (8.0%)
Unemployed	12 (3.7%)
Other	46 (14.2%)
**State of residence**	
South Darfur	276 (84.9%)
West Darfur	37 (11.4%)
North Darfur	5 (1.5%)
Unknown/unspecified	7 (2.2%)

### Patterns and Geographic Scope of Alleged Abuses

The attacks documented in the patients' records occurred between 2002 and 2006, with a peak frequency in March 2005. Characteristics of these incidents are displayed in Table 2. Between the date of the incident and the date of presentation at the Amel Centre, a median of 101 d had elapsed (inter-quartile range, 22–365 d). Approximately one-third (36.6%) of patients presented to the Amel Centre within 6 wk of the alleged incident.

**Table 2:** Characteristics of incidents that led to injuries.

Variable Name	Number (Percent) or Median (IQR)
**Incident year**	
2002	4 (1.2%)
2003	43 (13.2%)
2004	162 (50.0%)
2005	89 (27.4%)
2006	26 (8.0%)
Unknown/unspecified	1 (0.3%)
**Days elapsed between incident and presentation to Amel Centre**	101 (22–365)
**Days elapsed ≤42 d**	
Yes	119 (36.6%)
No	204 (62.8%)
Could not be calculated	2 (0.6%)
**Same incident also reported by another Amel Centre patient**	
Yes	114 (35.1%)
Unspecified	211 (64.9%)
**Rural council area where incident took place**	
Nyala	74 (22.8%)
Malam	59 (18.2%)
Abu Agura	34 (10.5%)
Yasin	32 (9.9%)
Shearia	32 (9.9%)
Other locations throughout North, South, and West Darfur	94 (28.9%)
Unknown	1 (0.3%)
**IDP camp where incident took place, if noted**	
Dreig	17 (37.0%)
Otash	15 (32.6%)
Kalma	14 (30.4%)
**Distance from IDP camp**	
Outside camp	21 (45.7%)
Inside camp	16 (34.8%)
In the general vicinity (but exact distance unspecified)	9 (19.6%)
**Distance outside IDP camp (kilometers)** a	3 (3–3)

a From the 15 records in which the patient provided an estimated distance to the examining clinician.

IQR, inter-quartile range.

**Figure 1:**
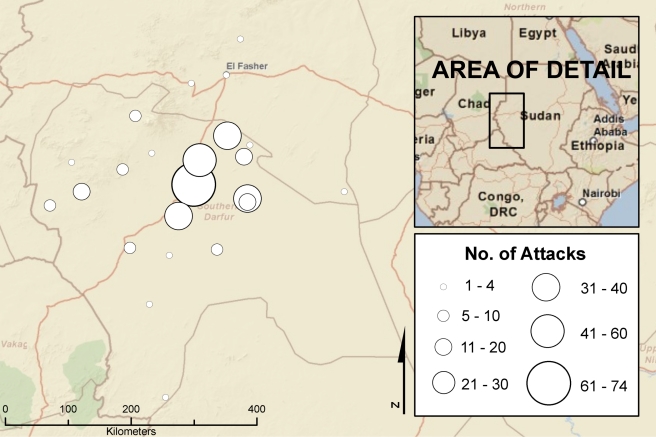
Geographic pattern of attacks reported by patients, 2002–2006. The largest circle corresponds to the town of Nyala, where the Amel Centre was located. The base map for this figure was obtained from ArcGIS (version 9.2, Esri) Online World StreetMap, accessed on February 22, 2011. Sources: Esri, DeLorme, NAVTEQ, TomTom, US Geological Survey, Intermap, Increment P Corporation, Natural Resources Canada, Esri Japan, and the Japanese Ministry of Economy, Trade and Industry.

Alleged attacks on individuals and villages recorded in the patients' records took place in 23 rural council areas (out of 65 total) throughout Darfur (Figure 1). Of the total, 281 (86.5%) occurred in South Darfur, 35 (10.8%) occurred in West Darfur, and eight (2.5%) occurred in North Darfur. Approximately one-third (35.1%) of the attacks disclosed by patients were also described by at least one other Amel Centre patient. Many villages were repeatedly attacked, with five villages reportedly attacked a total of 41 times during the study period: Marla was attacked 13 times during a 12-mo period from December 2004 to December 2005; Adwa, ten times (October 2003–November 2005); Labado, seven times (March 2004–December 2005); Bendisi, six times (August 2003–Dececember 2004); and Mukjar, five times (August 2003–August 2004). In addition, 46 (14.2%) patients disclosed that they had been attacked in the vicinity of an IDP camp: 16 (34.8%) of these attacks occurred inside the camp, 15 (32.6%) occurred a median of 3 km outside the camp, six (13.0%) occurred an unspecified distance outside the camp, and nine (19.6%) occurred within the general vicinity of a camp but the exact location was unspecified.

Two hundred ninety-three (90.1%) patients described their perpetrators as GoS and/or Janjaweed forces; of these, 48 (16.4%) stated that GoS and Janjaweed forces attacked in concert (Table 3). Thirty-two (9.9%) patients disclosed that they had been attacked by rebel soldiers, bandits, community authorities, or other community members. Among those attacked by GoS and/or Janjaweed, 292 (99.7%) patients were from 12 different non-Arabic-speaking tribes, and only one (0.3%) was from an Arab tribe. Thirty-two (9.9%) patients disclosed to the examining clinician that a military commander was present during the attack. Nearly all (98.8%) attacks occurred in the absence of active armed conflict between GoS/Janjaweed forces and rebel groups. The examining clinician noted when patients speculated as to reasons for the attack: 60 (18.5%) patients stated that they were targeted because the attackers suspected them of being rebels, and 58 (17.9%) stated that they were targeted because of their racial or tribal identity.

**Table 3:** Characteristics of alleged perpetrators.

Variable Name	Number (Percent) or Median (IQR)
**Affiliation of alleged direct perpetrator(s)**	
Janjaweed	166 (51.1%)
GoS	79 (24.3%)
Both GoS and Janjaweed	48 (14.8%)
Other	32 (9.9%)
**Military commander present**	
Yes	32 (9.9%)
Unspecified	293 (90.2%)
**Number of alleged perpetrators**	
Single perpetrator	7 (2.2%)
More than one but exact number unspecified	207 (63.7%)
2–5 perpetrators	55 (16.9%)
6–10 perpetrators	21 (6.5%)
More than ten perpetrators	35 (10.8%)
**Number of alleged perpetrators, if noted**	5 (2–20)
**Reason for incident as perceived by patient** a	
Suspected of being a rebel	60 (18.5%)
Targeted because of racial or tribal identity	58 (17.9%)
Suspected of supporting rebels	24 (7.4%)
Suspected of theft, or was defending self against theft	11 (3.4%)
Suspected of political activity	5 (1.5%)
Suspected of working against rebels	4 (1.2%)

a Responses in this category were not mutually exclusive, so percentages do not add up to 100.

IQR, inter-quartile range.

Patients' medical records described a wide range of alleged abuses, including beatings (161 [49.5%]), gunshot wounds (140 [43.1%]), destruction or theft of private property (121 [37.2%]), involuntary detainment (97 [29.9%]), and being bound with rope, chains, or other material (64 [19.7%]) (Table 4). GoS forces were described as accounting for more than one-half of custody-related incidents (61 [59.8%]), whereas Janjaweed forces were alleged to have accounted for most incidents involving physical assault (148 [50.7%]), sexual assault (28 [62.2%]), and destruction or theft of private property (77 [63.6%]). In addition to the abuses patients personally experienced, the medical records for this group of patients also collectively describe that they witnessed the killing of 948 other persons.

**Table 4:** Types of abuses disclosed by patients.

Type of Abuse	Affiliation of Alleged Perpetrator(s)	
	GoS and/or Janjaweed (*n* = 293)	Other/Unknown (*n* = 32)
**Attacks involving heavy weapons**	33 (11.3%)	2 (6.3%)
Ground explosives (bombing, grenades)	2 (0.7%)	
Attack by aircraft or helicopter	18 (6.1%)	1 (3.1%)
Attack by land cruiser	24 (8.2%)	1 (3.1%)
**Physical assault**	264 (90.1%)	28 (87.5%)
Blunt trauma (beating, whipping)	145 (49.5%)	16 (50.0%)
Gunshot wound	125 (42.7%)	15 (46.9%)
Burns or electric shocks	21 (7.2%)	
Stretch injury (hanging, suspension)	19 (6.5%)	
Genital trauma	10 (3.4%)	
**Sexual assault**	39 (13.3%)	6 (18.8%)
Forced undressing	12 (4.1%)	1 (3.1%)
Insertion of foreign object into anus/vagina	3 (1.0%)	
Attempted rape	5 (1.7%)	
Rape	15 (5.1%)	1 (3.1%)
Rape by more than a single perpetrator	12 (4.1%)	4 (12.5%)
**Humiliation or psychological manipulation**	70 (23.9%)	3 (9.4%)
Verbal abuse	32 (10.9%)	1 (3.1%)
Verbal abuse involving racial slurs	6 (2.1%)	
Forced performance of humiliating/taboo acts	7 (2.4%)	
Verbalized threats of death	43 (14.7%)	2 (6.3%)
**Custody-related violations**	95 (32.4%)	7 (21.9%)
Involuntary detainment	90 (30.7%)	7 (21.9%)
Bound with rope or other apparatus	60 (20.5%)	4 (12.5%)
Crowded, unhygienic conditions	43 (14.7%)	
Deprived of food/water or medical care	32 (10.9%)	1 (3.1%)
Sensory deprivation	25 (8.5%)	
**Destruction or theft of private property**	115 (39.3%)	6 (18.8%)

Data are number (percent).

### Consistency between Allegations of Abuse and the Signs and Symptoms Described in the Medical Records

The signs and symptoms most frequently documented in the medical records were chronic pain (194 [59.7%]), wounds or scars (167 [51.4%]), functional disabilities (e.g., contractures causing restricted grasp) (65 [20.0%]), and bone fractures (55 [16.9%]) (Table 5). There was 96.3% agreement (κ = 0.92) between the medical reviewers on whether the medical records contained sufficient detail to enable an informed opinion about the consistency of the recorded signs and symptoms with the allegations documented in the medical record. More than one-third (127 [39.1%]) of the medical records were assessed by at least one reviewer to lack sufficient detail (i.e., documentation was incomplete) to enable him or her to render an informed judgment about consistency. Of the 198 (60.9%) records that were considered sufficiently detailed by both reviewers, the medical reviewers agreed that the recorded signs and symptoms were either consistent with (101 [51.0%]), highly consistent with (81 [40.9%]), or virtually diagnostic of (5 [2.5%]) the alleged abuses. There were no cases in which the reports of medical examinations were considered not consistent with, or unrelated to, the recorded allegations. In only 11 (3.4%) cases did the medical reviewers disagree in their consistency scorings, for an excellent inter-rater agreement overall (κ = 0.89).

**Table 5:** Common symptoms and signs documented in patient medical records.

Type of Symptom or Sign	Number (Percent)
Pain (non-pelvic)	194 (59.7%)
Wounds or scars	167 (51.4%)
Functional disability	65 (20.0%)
Broken or fractured bones	55 (16.9%)
Weakness	38 (11.7%)
Pelvic pain	34 (10.5%)
Insomnia	32 (9.9%)
Numbness	23 (7.1%)
Swelling	18 (5.5%)
Headache	14 (4.3%)

Approximately one-half (36 [49.3%]) of all women presenting to the Amel Centre disclosed that they had been sexually assaulted. One-half of sexual assaults on women were recorded as having occurred in close proximity to an IDP camp, with nine (25.0%) recorded as having occurred in the general vicinity of the camp and nine (25.0%) having occurred within 3 km of the camp. The majority (31 [86.1%]) of sexual assaults on women involved rape or gang rape. Among these, five (16.1%) women disclosed they had become pregnant as a result of the alleged rape; no follow-up information was available on the remainder. Nine men also disclosed that they had been sexually assaulted, including one who had been raped. Twenty-five (55.6%) medical records of sexual assault victims were considered by the medical reviewers to be sufficiently detailed in recorded signs and symptoms to enable him or her to render an informed judgment about consistency. Of these, the medical reviewers agreed that the medical evidence was consistent with (14 [56.0%]), highly consistent with (9 [36.0%]), or virtually diagnostic of (1 [4.0%]) the alleged sexual assault. There were no cases in which the medical findings were considered not consistent with, or unrelated to, the alleged sexual assault. The reviewers disagreed about the scoring for one (4.0%) case, for an excellent inter-rater agreement on sexual assault findings overall (κ = 0.92).

## Discussion

We analyzed the medical records of 325 consecutive patients who were seen for care at the Amel Centre in Nyala, Darfur, between September 28, 2004, and December 31, 2006, with the aim of assessing the consistency between the recorded allegations of abuse and the signs and symptoms noted in the medical record. Our findings show that in all of the medical records that contained sufficient detail, the medical evidence was considered to be at least consistent with (if not highly consistent with or virtually diagnostic of) the human rights violations disclosed by the patients. There were no cases in which the reports of medical examinations were considered not consistent with, or unrelated to, the recorded allegations. Most of the abuses described in the medical records—which included beatings, killings, sexual assault, torture, and involuntary detainment—were allegedly perpetrated by GoS and Janjaweed forces and were described as having occurred throughout Darfur, with five villages attacked a total of 41 times during the study period. The spatial distribution of reported incident locations in our data suggests, at a minimum, that the attacks were widespread. However, our lack of a representative population-based sample makes it difficult for us to generalize about the full extent or population incidence of attacks. Many patients reported attacks by GoS and Janjaweed forces acting in concert. In some cases, patients disclosed to the examining clinician the names of specific victims, perpetrators, or military commanders, and this information was noted in the medical record. Fewer than 1% of patients reported observing the perpetrators to be in active armed conflict with rebel or other groups. Although the medical reviewers had no way to corroborate the identities of the perpetrators, these findings are consistent with prior research implicating GoS forces in the perpetration of human rights violations upon non-Arabic-speaking civilians in Darfur [24],[25].

Nearly one-half of women presenting for care disclosed that they had been sexually assaulted. The use of sexual violence in armed conflict has been recognized as a means of not only demoralizing individual victims but also destabilizing their families and terrorizing communities [51]–[56]. Rape and other forms of sexual violence have been recognized as war crimes and crimes against humanity [57], as well as instruments of genocide [55],[58]. Moreover, one-half of these assaults were described as having occurred in close proximity to an IDP camp. These data are consistent with prior reports of rapes occurring near IDP camps [26],[55],[59], as well as previous work documenting that violence was responsible for a substantial proportion of deaths among persons settled (i.e., not in the villages or in flight) in IDP camps in West Darfur [11]. Collectively, these data raise questions about the security provided to persons living in IDP camps, notably women, who must frequently venture outside the camp to gather firewood for fuel [27]. The Inter-Agency Standing Committee has issued guidelines that suggest several minimum prevention and response interventions that could be implemented with regards to security mechanisms instituted in areas of close proximity to IDP camps.

In contrast to prior studies' reliance on self-report of refugees living outside of Darfur [17],[24],[25],[27],[28], our data are based on unusual access to medical records of clinical encounters in Darfur maintained by local clinicians directly responsible for treatment and record-keeping. Medical forensic experts reviewed and analyzed the signs and symptoms described in the medical records and evaluated their consistency with the alleged abuses documented in the medical notes. Less than two-thirds of the records were detailed enough for the forensic reviewers to substantiate the patients' claims of abuse, a finding that is not surprising given that the Amel Centre medical records were not intended for research purposes. In a similar study in which third-party experts assessed the official medical evaluations of forensic experts working for the Mexican Procuraduría General de la República (Office of the Attorney General), in 18 of 39 cases (46%) their assessments were indeterminate because of insufficient documentation to corroborate alleged torture and ill treatment [60]. Their findings are consistent with ours and highlight the potential value of using clinical information to corroborate allegations of abuse. In our study, among the medical records that contained sufficient detail, all were assessed to be at least consistent with (if not highly consistent with or virtually diagnostic of) the allegations. These data substantially enhance the credibility of the patients' claims of abuse. Importantly, however, the medical records provided the forensic reviewers with no data that could be used to corroborate either claims of assailant identities or claims of genocidal intent.

### Limitations

Interpretation of our findings is subject to a number of limitations. First, we used a discrete, comprehensive sample of patients, but it was not systematic. During this time period, the Sudanese Criminal Procedure Act required all injury or trauma victims to file a report with the police in order to obtain a medical evidence form (“Form 8”), without which they were legally not permitted to receive treatment from an authorized medical officer [55],[61]–[63]. In practice the police were known to deny the Form 8 to members of non-Arabic-speaking tribes, so this legal requirement represented a substantial hurdle, and in many cases a complete barrier, to accessing health care services. Consistent with this, patients in our sample presented for care a median of 101 d after the abuses leading to their need for treatment. Furthermore, the majority of patients seen were men, highlighting the issue of women's lack of adequate access to care and their overall limited public mobility in this setting. These barriers are of particular salience with regards to cases of sexual violence [61], where victims may fear reprisals, blame, and other psychosocial consequences of disclosure [5],[26],[59],[64]–[68]. A second limitation relates to the delay in presentation for care. Although physical and psychological sequelae may persist for years and even for the duration of a victim's lifetime, some symptoms and disabilities may resolve or diminish over time [42]–[45],[69]. Despite their training and experience, Amel Centre staff could have under-detected and therefore under-documented some symptoms, especially those concerning sensitive topics (e.g., sexual violence, psychological distress) that might not be spontaneously disclosed. More generally, the medical chart review literature is characterized by under-documentation of signs and symptoms [70]–[73], so we would expect this limitation to generically apply in any setting. Third, because these data were not collected in a research setting, in most if not all cases, the same individual documented both the allegations of abuse and the results of the medical examination. The examining clinician's prior knowledge of the nature of the allegations could have biased the completeness of the documentation with regards to signs and symptoms observed. Fourth, few rape cases were scored by our medical forensic experts as virtually diagnostic of the alleged assault. Amel Centre protocol directed all female rape victims to a gynecologist for evaluation [74]. However, these records were unavailable for analysis because they could not be secured and sent out of the country prior to the clinicians' fleeing the country (as described above). Fifth, we were unable to include information on victims who were killed, so it may be more appropriate to regard our data as underestimating the true severity of atrocities inflicted upon non-Arabic-speaking civilians living in this region. Sixth, Amel Centre staff were routinely subject to surveillance, detainment, and interrogation by GoS forces [75],[76]. With increasing frequency in 2009, Amel Centre staff were detained, interrogated, tortured, and accused of collaborating with the ICC. Upon Al Bashir's indictment, they were advised to flee the country. Because of ongoing security concerns, we could not obtain the records for 2007–2009 to analyze for this study. This limitation underscores that the Amel Centre clinicians provided medical and legal services under dangerous working conditions. Health care workers in other settings have faced similar challenges [77], further emphasizing the need for international support for the protection of health professionals working under similar circumstances.

In summary, despite these unavoidable limitations, our study of non-Arabic-speaking civilian patients who visited the Amel Centre in Nyala, Darfur, between 2004 and 2006 found that in all of the medical records that contained sufficient detail, the recorded medical evidence was considered at least consistent with the alleged incidents of torture and other human rights violations. There were no cases in which the reports of medical examinations were considered not consistent with, or unrelated to, the recorded allegations. The widespread, organized, and sustained pattern of attacks documented in our study indicates that the actions of Janjaweed and GoS forces may constitute war crimes, crimes against humanity, and/or possibly acts of genocide.

## Supporting Information

Text S1 **Coding sheet, with lists of standardized names and response options, used to guide abstraction of data from the medical records (version of December 7, 2010).**
(PDF)

## Abbreviations

ICC: International Criminal Court
IDP: internally displaced person
GoS: Government of Sudan
