# Supplementary material for: Medical Evidence of Human Rights Violations against Non-Arabic-Speaking Civilians in Darfur: A Cross-Sectional Study
Source: PLoS Med. 2012 Apr 3;9(4):e1001198. doi: 10.1371/journal.pmed.1001198 (PMC3317898; doi:10.1371/journal.pmed.1001198)
Supplement: Text S1 — Coding sheet, with lists of standardized names and response options, used to guide abstraction of data from the medical records (version of December 7, 2010). (PDF) [file pmed.1001198.s001.pdf]

Text S1. Supporting Information. This coding sheet, with lists of standardized names and response options, was used to guide abstraction of data from the medical records (version Dec 7, 2010).

Socio-Demographic Variables

```
1.  Participant ID
2.  Sex:                                  0 Male          1 Female
3.  Pregnant at the time of the attack:   0 No            1 Yes           -99 N/A (for men)
4.  Day of reporting to the center (1-31)
5.  Month of reporting to the center (1-12)
6.  Year of reporting to the center (2004-2006)
7.  Age (years)
8.  Marital status:       0 Single              1 Married      Free text: specify other
9.  Number of wives       -88 more than one but exact # unspecified
                          -99 N/A (for women)
10. Number of children    -88 more than one but exact # unspecified
11. Number of sons
12. Religion:             1 Muslim
                          Free text: specify other religion
13. Tribe/Ethnicity:        1 Zaghawa
                            2 Masalit (Massaleit)
                            3 Fur
                            4 Dago (Dajo)
                            5 Meisseiria (Missiriya)
                            6 Tamma
                            7 Burno (Borno, Bornu)
                            8 Erenga
                            9 Tunjur
                           10 Habbaniya (Habaniya)
                           11 Rizeigat (Rizaygat, Rezeqat)
                           12 Fallata (Fulani)
                           13 Beni Halba (Bani Halba)
                           14 Ta'isha (Ta'aisha)
                           15 Tarjum
                           16 Hawara
                           17 Bargo
                           18 Humur (Hammer)
                           19 Marareit
                           20 Burti
                           21 Kamalti
                           22 Meidoub
                           23 Gimmir
                           24 Shaigyya (Shaigiya)
                           25 Jaalieen
                           26 Danagila (Dangila)
                          Free text: specify other tribe
14. Occupation:           1 Farmer
                          2 Merchant
                          3 Student/Pupil
                          4 Shepherd
                          5 Teacher
                          Free text: specify other occupation
15. Place of residence:   1 South Darfur
                          2 West Darfur
                          3 North Darfur
                          Free text: specify if other residence
16. Political affiliation (free text)
```

Characteristics of Incident that Led to Reported Injury

17. Day of incident (1-31): *leave blank if day not indicated*
18. Month of incident (1-12): *leave blank if month not indicated*
19. Year of incident (2004-2006)
20. Time of incident (12-hour clock): *leave blank if exact time not indicated*
21. Time period of incident: 1 Ante meridiem A.M.
    2 Post meridiem P.M.
22. Location of incident: (Rural Council Boundaries)

North Darfur
1 Malha
2 Malit & Sayeh
3 Korni, Um Baru & Tina
4 Kutum Rural & Fato Borno
5 Al Sireaf & Saraf Omra
6 Kabkabiya & Jebel Si
7 Tawila & Korma
8 Al Fasher & Kuma
9 Dar al Salam
10 Um Kadada
11 Al Tawisha
12 Al Lait

West Darfur
13 Kulbus
14 Seleia
15 Sirba
16 Kerenek
17 Masteri
18 Mornei
19 Beida
20 Habilah
21 For Baranga
22 Azoum
23 Abata
24 Zallingi Rural - Traige
25 Um Kher
26 Garseila-Delaige
27 Bundis
28 Mukjar
29 Um Dukhun

South Darfur
30 Kass
31 Shataya
32 Sharq Gabal Mara
33 Malam
34 Abu Agura
35 Nyala
36 Balil
37 Yasin
38 Mahagriya
39 Shearia
40 Natiqa
41 Al Deain
42 Fardus
43 Asalaya
44 Abu Matariq

45 Abu Gabra
46 Adayla
47 Abu Karaynka
48 Kubum
49 Um Lubasa
50 Edd Al Fursan
51 Madkandi
52 Katayla
53 Antikayna
54 Tulus
55 Damsu
56 Nyimeri
57 Um Dafug
58 Rehed al Birdi
59 Girayda
60 Buram
61 Wad al-Ugam
62 Suntah-al
63 Guhayna-al
64 Guz-al
65 Radum-al
Free text: other location not specified in this list

23. Number of alleged perpetrators: *enter a number from 1-999*
-88 more than one but exact # unspecified

24\. Affiliation of alleged perpetrators:
1 Janjaweed
3 Both Janjaweed and Sudanese government
4 Rebel soldier
5 Bandit (i.e., no specific affiliation identified)
6 Community authority
7 Community member (e.g., neighbor)
8 Other
-88 unknown or not specified

25\. Military commander present:
0 No
1 Yes
-88 Unknown

26\. Name of alleged perpetrator 1: *(spell name, rank)*
27\. Name of alleged perpetrator 2: *(spell name, rank)*
28\. Name of alleged perpetrator 3: *(spell name, rank)*
29\. Name of alleged perpetrator 4: *(spell name, rank)*
30\. Name of alleged perpetrator 5: *(spell name, rank)*
31\. Name of alleged perpetrator 6: *(spell name, rank)*
32\. Name of alleged perpetrator 7: *(spell name, rank)*
33\. Name of alleged perpetrator 8: *(spell name, rank)*
34\. Name of alleged perpetrator 9: *(spell name, rank)*
35\. Name of alleged perpetrator 10: *(spell name, rank)*
36\. Alleged perpetrator(s) reported to be defending themselves against attack 0 No 1 Yes
37\. Alleged perpetrator(s) reported to be in armed conflict w/ militants 0 No 1 Yes
38\. Alleged perpetrator(s) reported to be intoxicated 0 No 1 Yes

Types of Abuses Experienced Personally by Client

39\. Violence *personally* experienced by client 0 No 1 Yes

Physical Violence

40\. Blunt trauma: beating, whipping, slapping
41\. Gunshot wound
42\. Ground explosives: bombing, grenades, shrapnel
43\. Aerial bombing
44\. Other penetrating or perforating trauma: knife, spear

45. Crush injuries: smashing fingers
46. Extraction/amputation injuries: removal of fingers/teeth
47. Burns: cigarettes, heated instruments, scalding liquid
48. Electric shocks
49. Suffocation/asphyxiation: wet/dry methods, drowning, choking, chemical
50. Hanging/stretching/suspension
51. Chemical exposure: salt, chili pepper, gasoline, drugs

Sexual Violence

52. Unwanted sexual advances
53. Forced to undress
54. Unwanted touching: touching, kissing, grabbing, fondling
55. Insertion of foreign object into vagina/rectum
56. Attempted rape: assailant attempted rape but did not succeed
57. Rape (either anal or vaginal)
58. Gang rape: more than one assailant
59. (If gang rape): number of assailants: *enter a number from 1-99*
    -88 more than one but exact # unspecified
60. Forced prostitution

Psychological Manipulations

61. Verbal abuse, including mockery
62. Verbal abuse characterized by racial/ethnic slurs
63. Threats of sexual violence
64. Threats of physical torture/beating
65. Threats against family
66. Threats of death
67. Performance of humiliating acts, violation of taboos (e.g., head shaven with unclean razor)
68. Forced witnessing of torture/atrocities inflicted on others

Detainment

69. Detained against will: kidnapped, held captive
70. Duration of detainment (in days) -- use fractions for hours
71. Forced to serve in military forces

Conditions of Detainment

72. Bound with rope, chains, or other restriction of movement
73. Forced standing
74. Small or overcrowded cell
75. Solitary confinement
76. Unhygienic conditions, infested surroundings
77. No access to toilet/bathing facilities
78. Cold showers, doused with cold water
79. Deprived of food
80. Deprived of water
81. Deprived of medical care
82. Sensory deprivation (e.g., complete darkness)
83. Exposure to extreme heat or cold
84. Exposure to bright light
85. Exposure to loud music

Material Loss

86. Destruction of crops
87. Destruction of home
88. Theft of livestock
89. Killing of livestock
90. Other type of violence experienced not specified (free text)
91. Was incident observed by other persons? 0 No
    1 Yes
    -88 Unknown/not specified

92. Narrative passage describing violence experienced by client (free text)

Types of Abuses Witnessed by Client

93. Violence was witnessed by client: specify number of victims, 1-999
    -88 more than one but exact # unspecified
    Free text: specify other ("many people")
94. Was the witnessed violence associated with the primary event? 0 No
    1 Yes

    Physical Violence
95. Blunt trauma: beating, whipping, slapping
96. Gunshot wound
97. Ground explosives: bombing, grenades, shrapnel
98. Aerial bombing
99. Other penetrating or perforating trauma: knife, spear
100. Crush injuries: smashing fingers
101. Extraction/amputation injuries: removal of fingers/teeth
102. Burns: cigarettes, heated instruments, scalding liquid
103. Electric shocks
104. Suffocation/asphyxiation: wet/dry methods, drowning, choking, chemical
105. Hanging/stretching/suspension
106. Chemical exposure: salt, chili pepper, gasoline, drugs
107. Wilful killing (according to any of the above methods)

    Sexual Violence
108. Unwanted sexual advances
109. Forced to undress
110. Unwanted touching: touching, kissing, grabbing, fondling
111. Insertion of foreign object into vagina/rectum
112. Attempted rape: assailant attempted rape but did not succeed
113. Rape (either anal or vaginal)
114. Gang rape: more than one assailant
115. (If gang rape): number of assailants: enter a number from 1-99
    -88 more than one but exact # unspecified
116. Forced prostitution

    Psychological Manipulations
117. Verbal abuse, including mockery
118. Verbal abuse characterized by racial/ethnic slurs
119. Threats of sexual violence
120. Threats of physical torture/beating
121. Threats against family
122. Threats of death
123. Performance of humiliating acts, violation of taboos (e.g., head shaven with unclean razor)
124. Forced witnessing of torture/atrocities inflicted on others

Detainment
125. Detained against will: kidnapped, held captive
126. Duration of detainment (in days) -- use fractions for hours
127. Forced to serve in military forces

Conditions of Detainment
128. Bound with rope, chains, or other restriction of movement
129. Forced standing
130. Small or overcrowded cell
131. Solitary confinement
132. Unhygienic conditions, infested surroundings
133. No access to toilet/bathing facilities
134. Cold showers, doused with cold water

135. Deprived of food
136. Deprived of water
137. Deprived of medical care
138. Sensory deprivation (e.g., complete darkness)
139. Exposure to extreme heat or cold
140. Exposure to bright light
141. Exposure to loud music

Material Loss
142. Destruction of crops
143. Destruction of home
144. Theft of livestock
145. Killing of livestock
146. Other type of violence witnessed not specified (free text)
147. Narrative passage describing violence witnessed by client (free text)

Other Miscellaneous Data

148. Perceived reason for violence: Suspicion of being a rebel	0 No	1 Yes
149. Perceived reason for violence: Suspicion of being a rebel supporter	0 No	1 Yes
150. Perceived reason for violence: Suspicion of political activity	0 No	1 Yes
151. Perceived reason for violence: Suspicion of being a traitor	0 No	1 Yes
152. Perceived reason for violence: Race/tribe/religion	0 No	1 Yes
153. Perceived reason for violence: Other (free text)		
154. Previous arrest/detention:	0 No	1 Yes
155. Prior torture/mistreatment:	0 No	1 Yes

156. How client came to Amel Center:
- 1 Self-referral
- 2 Referred by friend/relative
- 3 Brought to center by friend/relative
- 4 Brought to center by staff
- 5 Other

157. Assistance: Medical care	0 No	1 Yes
158. Assistance: Legal services	0 No	1 Yes
159. Referral: Psychiatry	0 No	1 Yes
160. Referral: Gynecology	0 No	1 Yes

Sequelae of Incident

161. Hospitalization required:	0 No	1 Yes
162. Symptoms: Chronic (non-pelvic) Pain	0 No	1 Yes
163. Symptoms: Chronic Pelvic Pain	0 No	1 Yes
164. Symptoms: Headache	0 No	1 Yes
165. Symptoms: Problems with vision	0 No	1 Yes
166. Symptoms: Problems with hearing	0 No	1 Yes
167. Symptoms: Numbness	0 No	1 Yes
168. Symptoms: Weakness	0 No	1 Yes
169. Symptoms: Insomnia	0 No	1 Yes
170. Symptoms: Hallucinations	0 No	1 Yes
171. Symptoms: Depressed mood	0 No	1 Yes
172. Symptoms: Flashbacks	0 No	1 Yes
173. Symptoms: Other (free text)		
174. Signs: Wounds or scars	0 No	1 Yes
175. Signs: Swelling	0 No	1 Yes
176. Signs: Decreased visual acuity	0 No	1 Yes
177. Signs: Hearing loss/impairment	0 No	1 Yes
178. Signs: Peripheral numbness	0 No	1 Yes
179. Signs: Tremor	0 No	1 Yes
180. Signs: Broken or fractured bones	0 No	1 Yes
181. Signs: Functional disability	0 No	1 Yes
182. Signs: Sexually transmitted disease	0 No	1 Yes
183. Signs: Genital trauma	0 No	1 Yes
184. Signs: Other (free text)		

(If client was exposed to sexual violence)

185. (If not previously with child) Became pregnant: 0 No 1 Yes -88 Unknown
186. (If previously with child) Miscarriage or abortion: 0 No 1 Yes

Prognosis, as Determined by Treating Clinician (CHOOSE ONE ONLY)

187. Full recovery: 0 No 1 Yes
188. Partial recovery with residual physical disability: 0 No 1 Yes
189. Partial recovery with residual psychiatric disability: 0 No 1 Yes

Medical Evaluation of Signs and Symptoms

CLINICAN: Please review the medical record and then come to an overall assessment to answer the following two questions:

190. Does the medical record contain a level of detail sufficient to enable you to determine whether the documented signs and symptoms are consistent with the allegations of abuse?

    Yes
    No → Skip to end

191. (If the medical record contains sufficient detail) How consistent are the signs and symptoms with the allegations of abuse?

    Not Related to Alleged Abuse (NR)
    Not Consistent With Alleged Abuse (NCW)
    Consistent With Alleged Abuse (CW)
    Highly Consistent With Alleged Abuse (HCW)
    Virtually Diagnostic Of Alleged Abuse (VDO)
